# Effect of integration of poultry manure and vinasse on the abundance and diversity of soil fauna, soil fertility index, and barley (*Hordeum aestivum* L.) growth in calcareous soils

**DOI:** 10.1186/s12870-022-03881-6

**Published:** 2022-10-19

**Authors:** Mostafa Seleem, Naglaa Khalafallah, Raghda Zuhair, Adel M. Ghoneim, Mahmoud El-Sharkawy, Esawy Mahmoud

**Affiliations:** 1 Department of Soil and Water Science, Faculty of Agriculture, Tanta University, Tanta, Egypt; 2 Department of Zoology, Faculty of Science, Tanta University, Tanta, Egypt; 3 Agricultural Research Center, Field Crops Research Institute, 12112 Giza, Egypt

**Keywords:** Calcareous soils, Soil fauna, Poultry manure, Vinasse, Organic amendments, Barley plant

## Abstract

**Background:**

In Egypt, calcareous soils represent a large part of desert soils suffering from a shortage of nutrients and organic matter, affecting production and biological diversity in agroecosystems. Organic wastes, negatively affect the environment, recycling it as a promising technology in different farming systems, and its impact on crop productivity and soil fauna is largely unknown. In this study, the effects of integrating poultry manure (PM) alone or combined with vinasse (V) at rates of 4.2 g kg^− 1^ and 6.3 g kg^− 1^ in pots, on improving soil fauna diversity, soil fertility, soil consistency and yield of barley (*Hordeum aestivum* L.) grown in a calcareous soil were investigated.

**Results:**

The results showed that the addition of PM alone or combined with V at different rates led to a significant increase in the microbial biomass carbon (MBC), organic matter (OM), NPK soil availability and yield of barley. The addition of 6.3 g PM and 4.2 g V kg^− 1^ soil have the best results in OM with 65.0% compared to control, and V contributes more than 16% of them. Prostigmata and Collembola were the dominant groups and accounted for 43.3% and 50.0% in the PM1 and 50.0% and 20.0% in the PM2 of the total individuals, respectively. Shannon and Evenness indices increased significantly with the soil amended by PM alone or combined with V. Soil fauna plays a key role in soil consistency because of a significant relationship between soil fauna and soil OM, MBC and soil fertility index. The addition of 6.3 g PM and 4.2 g V kg^− 1^ soil gave the best results in grain yield by 76.90% compared to the control.

**Conclusion:**

In conclusion, the interaction between PM and V can be used as a promising organic amendments to increase barley yield and improve efficiency of a recycled PM and V on soil fauna and soil fertility of calcareous soil.

**Supplementary Information:**

The online version contains supplementary material available at 10.1186/s12870-022-03881-6.

## Background

In Egypt, calcareous soils are estimated at about 0.28 million hectares, which represents a large part of the desert land, especially in the northwestern coastal region and Sinai [1]. Calcareous lands are known as having a high content of calcium carbonate and high pH, which affects their chemical, biological, environmental and physical properties and thus is reflected in the cropping pattern and productivity [2]. Calcareous soil lacks organic matter (OM), whose addition in the form of compost or poultry manure, etc., improves the chemical, ecological and physical properties and increases the productivity of crops. The reuse of agricultural organic waste enhances the protection of natural resources and helps in the recycling of carbon and other mineral elements. When used judiciously, using this waste can improve soil quality, increase biota, and reduce reliance on commercial fertilizers that are considered hazardous [3, 4].

Sugar and Integrated Industries Company in Hawamdiya produces more than 2,000 cubic meters per day of vinasse, which is harmful to the environment, causing pollution to the waterways that drain it [5]. A litre of ethanol produces an average of 10 to 15 L of vinasse and is a waste liquid produced during the production of ethanol from sugarcane or sugar beet or cellulosic material. Vinasse is a low-cost alternative to fertilization because it replaces fertilizers and improves crop productivity through improving the soil structure [6]. Vinasse enhances soil fauna and microbial communities [7] and increases soil fertility, soil quality and crop production [6, 8]. Pinto et al. [9] found that the application of vinasse for two years improves the biological and chemical properties of the soil and increases the yield of corn, soybeans and pastures.

Poultry manure is an excellent soil amendment that provides nutrients for growing crops when used wisely, because it is high in organic matter along with nutrients available for plant growth [10]. Blay et al. [11] found the possibility of using poultry manure to improve burnt soil and increase rye-grass yield. The decomposition of organic waste is one of the most important biochemical processes, which is due to the biological activity in the soil, which increases its fertility [12]. The use of poultry manure (30 Mg of dry matter ha^− 1^) significantly improved the stability of mounds and increased the yield of many grasses and legumes [13]. Moreover, poultry manure was the best organic waste for the reclamation of burnt soils [14]. Castro et al. [15] found the possibility of using poultry manure to improve burnt soil and increase rye-grass yield. The application of seed endophytic microbial consortium as bio-fertilizer helps to increase soil health and soil biodiversity, improved chickpea yield, and reduce the use of chemical fertilizers that are harmful to the environment [16, 17].

Biodiversity in agricultural systems is a promising ecological strategy for achieving sustainable production. Residue recycling is a key factor in determining soil bioactivity and the managing fauna diversity of species in agro-ecosystems [18]. Soil fauna play an important role in changing soil quality by improving soil structure, decomposing organic matter, and increasing soil fertility and the number of organisms [19]. Soil fauna, which is part of Eucaryota, are grouped into the macrofauna, mesofauna, and microfauna [20]. These soil organisms contribute to the maintenance and productivity of ecosystems by affecting soil quality and health. Among the mesofauna organisms, Collembola contributes significantly to the addition of soil nutrients through the decomposition of organic matter [21]. Collembolans known to provide a healthy soil environment in the ecosystem by recycling and decomposing agricultural waste in the soil, thus they are environmentally friendly and also host many pathogens like fungi, bacteria, nematodes, protozoa, etc. [22]. Orbatid mites constitute one of the richest groups of arthropods in soil. Orbatid mites play an important role in the biogene stage of plant residues humification, and can react very sensitively to changes in natural and agricultural soils, and thus are used as bioindicators of the soil in which they live [19]. Barley is the fourth most important food crop in Egypt, with a cultivated area of about 126,000 hectares in 2020 that produced 356,580 tons with an average of 2830 kg ha^− 1^ [23]. Barley (*Hordeum aestivum* L.) plants are commonly grown in newly reclaimed soils (calcareous soils) which are generally characterized by low fertility, high calcium carbonate content and high pH [24]. Organic amendments play a positive role in improving soil fertility by increasing organic carbon, organic nitrogen, microbial biomass and enzymatic activity. Organic amendments largely contribute to stimulating soil fauna abundance with increasing nutrient cycling in sandy loam soils, while organic amendments serve as a nutrient source for crop production in sandy loam soils [25]. Organic amendments play an important role in increasing soil fertility and soil fauna for crops grown on calcareous soils. Therefore, the present study aims to explore the effects of integrating PM with V on the abundance and diversity of soil fauna and barley growth (*Hordeum aestivum* L.) grown in calcareous soil.

## Materials and methods

### Soil and amendments

Soil samples (0–50 cm depth) used in this experiment was collected and mixed from the Qetaa Maryout area (latitude 30.88°86°N, 29,8°54°E) Amreya 1, Alexandria Governorate, Egypt. Two types of organic amendments were used (Poultry manure (PM) and vinasse (V)) in this study. The Poultry manure was collected from a poultry farm using sawdust in the litter poultry for 40 days. The vinasse, a by-product was obtained from Egyptian sugar & integrated Industries Company, El Hawamdeyia (produces more than 2,000 m^3^ d ^− 1^ of vinasse), Cairo, Egypt. The properties of the soil, PM and V were determined according to Page et al. [26] and presented in (Table 1).

**Table 1:** Selected physicochemical properties of the soil, vinasse (V) and poultry manure (PM) used in this study

Characteristics	Units	Soil	V	PM
pH	-	7.88 ± 0.15 (1:2.5 extract)	4.00	8.16 ± 0.25 (1:10 extracts)
EC	dSm^− 1^	11.30 ± 0.05 (1:5 extract)	42.0	5.64 ± 0.07 (1:10 extracts)
Ca^2+^	meqL^− 1^	57.40 ± 1.25	290.0	155.0 ± 1.90
Mg^2+^	meqL^− 1^	51.48 ± 1.05	183.3	116.6 ± 1.63
O.M	%	0.85 ± 0.02	59.0	63.4 ± 2.05
CEC	cmol(+) kg^− 1^	10.6 ± 1.20	ND	ND
CaCO_3_	%	28.0 ± 1.24	ND	ND
Total N	%	0.098 ± 0.05	3.6	4.20 ± 0.15
Total P	%	0.05 ± 0.01	4.0	0.68 ± 0.08
Total K	%	0.43 ± 0.05	6.8	1.12 ± 0.65
Bulk density	g cm^− 3^	1.50 ± 0.1	1.06	0.6

ND, not determined; EC, electrical conductivity; OM., organic matter, and; CEC, cation exchange capacity. Results are expressed as mean ± standard deviation of five replicates

### Plant material

One cultivar of cultivated barley (*Hordeum aestivum* L.), variety 138 (origin, Egypt (2019); *rbc*L, MW391914; *mat*K GenBank, MW336989; Kind, Naked; Pedigree, /5/Aths/lignee686/3/Deir Alla106//Sv.Asa/Attiki/4/Cen/Bglo.”S”. It was obtained from Barley Research Department, Field Crops Research Institute, Sakha Station, and Agricultural Research Center, Egypt. The collection of barley cultivar used in this experiment complies with institutional, national and international guidelines and legislation.

### Experimental design

Barley (*Hordeum aestivum* L.) was grown in plastic pots, each containing 10 kg of soil samples under greenhouse conditions. The soil was air -dried, then crushed and sieved with a 4 mm sieve to reflect the natural conditions of the soil. The soil used was uniformly mixed with PM and V and their mixtures and filled into plastic pots of 30 cm in diameter and 23 cm in height (The height of the soil in the pots is 16 cm).

**Fig. 1:**
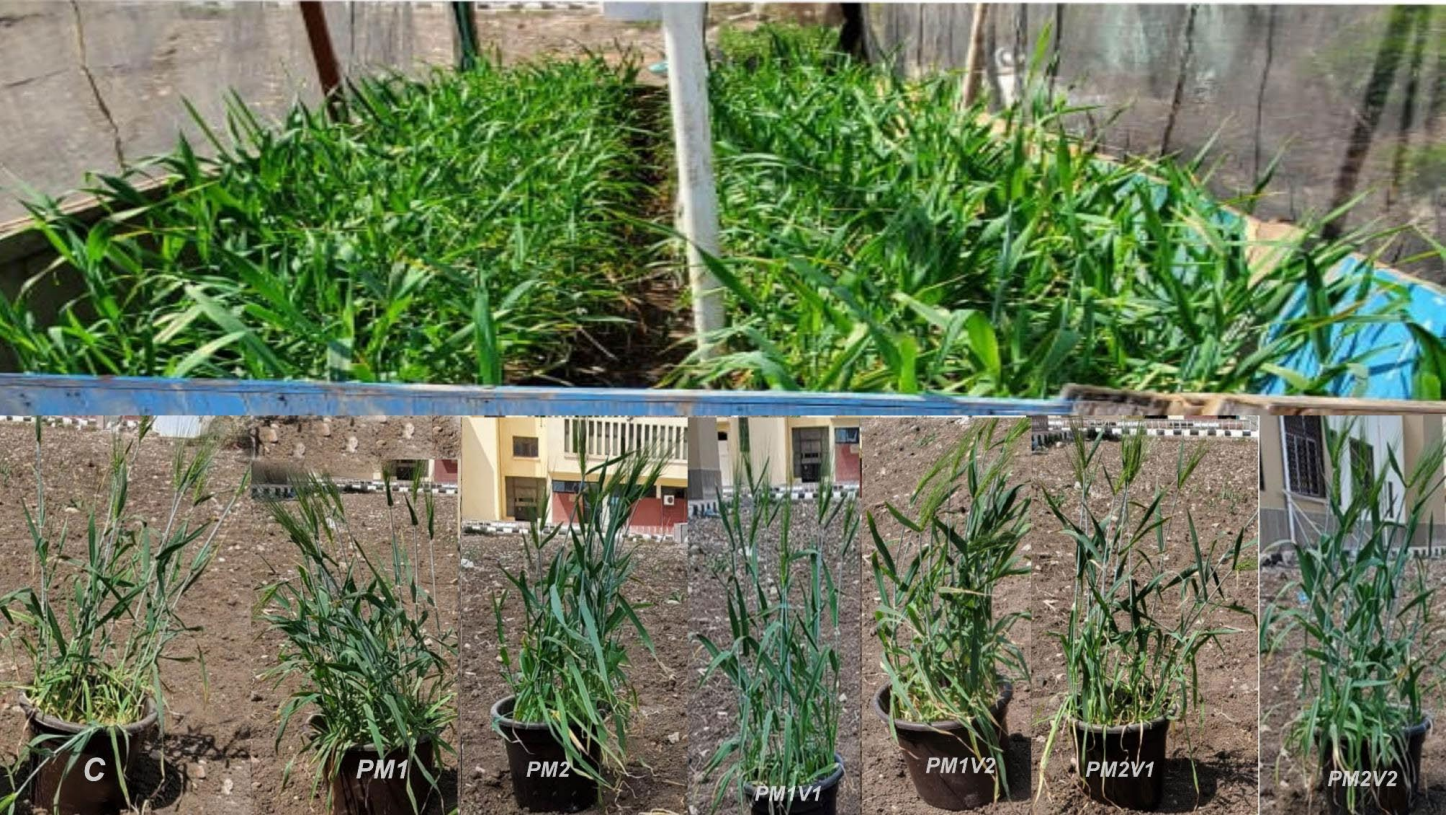
Experiment pots of barley plants after 125 days of sowing. Where C: Control; PM1: poultry manure at rate 10 t ha^− 1^; PM2: poultry manure at rate 15 t ha^− 1^; PM1V1: poultry manure at rate 10 ha^− 1^ + vinasse at rate 3.5 t ha^− 1^; PM1V2: poultry manure at rate 10 t ha^− 1^ + vinasse at rate 7 t ha^− 1^; PM2V1: poultry manure at rate 15 t ha^− 1^ + vinasse at rate 3.5 t ha^− 1^; PM2V2: poultry manure at rate 15 t ha^− 1^ + vinasse at rate 7 t ha^− 1^

Seven treatments were used in this experiment, and randomized complete blocks with five replicates were designed. The treatments used were: C: Control (NPK recommended doses); PM1: PM at 4.20 g kg^− 1^ soil; PM2: PM at 6.30 g kg^− 1^ soil; PM1V1: PM at 4.20 g kg^− 1^ soil + V at 4.2 g kg^− 1^ soil; PM1V2: PM at 4.20 g kg^− 1^ soil + V at 6.30 g kg^− 1^ soil; PM2V1: PM at 6.30 g kg^− 1^ soil + V at 4.20 g kg^− 1^ soil; and PM2V2: PM at 6.30 g kg^− 1^ soil + V at 6.30 g kg^− 1^, respectively. Seed barley (*Hordeum aestivum* L.) was planting (DAP) on 15 November 2021 and irrigated based on water requirements (based on field capacity) with 15% added as leaching requirements. Ten barley seeds were sown in each pot after 3 weeks thinning plants to five plants per pot. Recommended doses of NPK fertilization for barley according to the Egyptian Ministry of Agriculture were addition. Phosphorus was added at rate of 250 kg ha^− 1^ before cultivation as superphosphate 15.5% P_2_O_5_. Nitrogen doses were added in equal three times at rate 240 Kg ha^− 1^ as Urea 46%. Potassium was added as potassium sulphate 48% K_2_O at rate 120 kg ha^− 1^. All other agricultural practices on the experiment were done as recommended in the study area. The climatic conditions of the pot experiment were as follow: maximum and minimum temperature of 25–30 and 9–18^◦^C, relative humidity of 45–60%, and no rainfall during the experiment periods. Harvested 154 days after planting (DAP) on 3 May 2021, and yield measurements such as dry weight, 1000 grain weight, and grain weight of barley plant were recorded. Soil samples was collected from the rhizosphere area of barley plant for each pot soil and considered as a single replicate. After the soil was collected, it was immediately placed in a sterile plastic bag in an ice box to measure the some soil properties. Experiment pots of barley plants as shown in Fig. 1.

### Experimental analytical procedures

Organic matter of PM and V was determined by combustion method [26]. While, organic carbon of soil was determined by wet digestion process by 1 N potassium dichromate solution and sulphuric acid using Walkley and Black method, from where organic matter was calculated.

Organic matter (%) = organic carbon (%) ×1.724 [27].

pH and Electrical conductivity (EC) of soil, nB and nWTR were measured by using the pH meter and conductivity meter, respectively. Available P was determined using spectrophotometer using the ascorbic acid method after extracted by 0.5 M NaHCO_3_ solution at pH 8.30 according to Olsen et al. [28]. Available N (NH_4_^+^ and NO_3_^−^) was determined using the Kjeldahl method after extracted by 2 M KCl solution according to Soil Survey Staff [29]. Available K was determined using a flame photometer after extracted by 1.0 N ammonium acetate at pH 7 [26]. The properties of PM, V and the studied soils were analyzed according to standard methods [30]. Soil bulk density of the undisturbed soil sample was measured using the core method as explained by Grossman and Reinsch [31].

Microbial biomass carbon (MBC) was determined with 25 g of soil samples were fumigated with ethanol-free chloroform for 24 h at 25 ^0^ C [32]. Then the soil was extracted with K_2_SO_4_ and the extractable organic C was estimated using K_2_Cr_2_O_7_ and H_2_SO_4_ for 30 min at 170^o^C and titrated against ferrous ammonium sulphate with ferroin as the indicator. Microbial biomass carbon (MBC) was calculated from:

MBC = EC fumigated soil – EC un-fumigated soil/ Kc.

Where: EC = Extractable carbon.

Kc = 0.379 (Kc is the K_2_SO_4_ extract efficiency factor [33].

### Soil fauna

Soil samples were taken to determine the soil fauna at the mature stage (ripening stage) of barley growth (5 April 2021). Soil samples were collected using a metal core (10 cm x 10 cm), Micro-arthropods were extracted using a modified Berlese’s apparatus with a mesh size 1 mm. The extraction lasted 5 days. The organisms were collected in 70% ethanol. Adult oribatid mites were cleared with 70% lactic acid and identified to species level according to Balogh and Balogh [27], and check-list of Egyption oribatids [34, 35].

Species richness and Shannon–Wiener diversity index (Hˊ) and Pielou’s evenness (Jˊ) were calculated using PAST, a software package for paleontological data analysis V4.08 [36]. Shannon-Wiener index (H`) was calculated as -∑Pi ln (Pi), where Pi is the proportion of species i relative to the total number of species. Evenness (Jˊ) was calculated as Jˋ= Hˊ/lnS.

The fertility index was described according to Abdellatif et al. [37] with following equation:

$${\rm{FI}}\,{\rm{ = }}\,{\left( {{\rm{FN \times FP \times FK \times FOM}}} \right)^{1/5}}$$

Where: FI = fertility index; FN, FP, FK = available of nitrogen, phosphorus, potassium, respectively; and FOM = organic matter (%).

### Statistical analyses

All data are the means of 5 replicates. Normality of the data was tested with the Kolmogorov-Smirnov test. One–way analysis of variance (ANOVA) was applied to determine the significant differences among different treatments and Duncan multiple post hoc test. The values of p ≤ 0.05 were considered statistically significant. Data presented mean ± standard deviation (SD).The obtained data were statistically analyzed using program of SAS 9.4 (SAS, institute Inc., Cary, NC).

## Results

### Effect of poultry manure and vinasse on the abundance and diversity of soil fauna

The addition of PM alone or combined with V had a significant effect on the soil fauna (Table 2). Total individuals increased by 106.3%, 243.8%, 118.8% and 43.8% in PM1V1, PM1V2, PM2V1 and PM2V2 relative to control, respectively. Prostigmata and Collembola were the dominant groups and accounted for 43.3% and 50.0% in the PM1 and 50.0% and 20.0% in the PM2 of the total individuals, respectively. The highest mean numbers of soil collembola were found in PM1V1 pots, followed by treatments with PM1V2, PM2V1 and PM2V2 and the lowest abundant in the control. PM1V2 mixtures gave the best results in prostigmata and Oribatid mites by 580% and 133.3%, compared to the control, respectively. The combination of PM and V increased the numbers of soil prostigmata per pot from 1.25 to 5.0 in PM1V1 and to 8.5 in PM1V2.

**Table 2:** Effect of PM alone or co-applied with V on number of soil fauna in calcareous soils. Results are expressed as mean ± standard deviation of five replicates

Treatments	Prostigmata	Mesostigmata	orbatid mites	Collembola	Others	Total
C	1.25 ± 0.96 ^g^	0.25 ± 0.50^d^	0.75 ± 0.50^c^	1.00 ± 1.41^e^	0.75 ± 0.96^b^	4.00 ± 0.82^f^
PM1	3.25 ± 2.06 ^e^	0.00 ± 0.00^e^	0.25 ± 0.50^e^	3.75 ± 2.06 ^a^	0.25 ± 0.5^d^	7.50 ± 3.87^d^
PM2	3.75 ± 1.50^d^	1.25 ± 0.50^a^	0.50 ± 1.50^d^	1.5 ± 1.71 ^c^	0.50 ± 0.50^c^	7.50 ± 3.86^d^
PM1V1	5.00 ± 2.71^b^	0.75 ± 0.96^c^	0.50 ± 1.00^d^	1.75 ± 2.06 ^b^	0.25 ± 0.50^d^	8.25 ± 4.04^c^
PM1V2	8.5 ± 6.76^a^	1.00 ± 0.82^b^	1.75 ± 2.06^a^	1.25 ± 1.26^d^	1.25 ± 1.50^a^	13.75 ± 8.42^a^
PM2V1	4.50 ± 1.73 ^c^	1.25 ± 1.50^a^	1.00 ± 1.41^b^	1.25 ± 1.89^d^	0.75 ± 0.96^b^	8.75 ± 1.89 ^b^
PM2V2	3.00 ± 2.16^f^	0.25 ± 0.50^d^	0.75 ± 0.96^c^	1.25 ± 2.50^d^	0.50 ± 1.00^c^	5.75 ± 4.11^e^

**Fig. 2:**
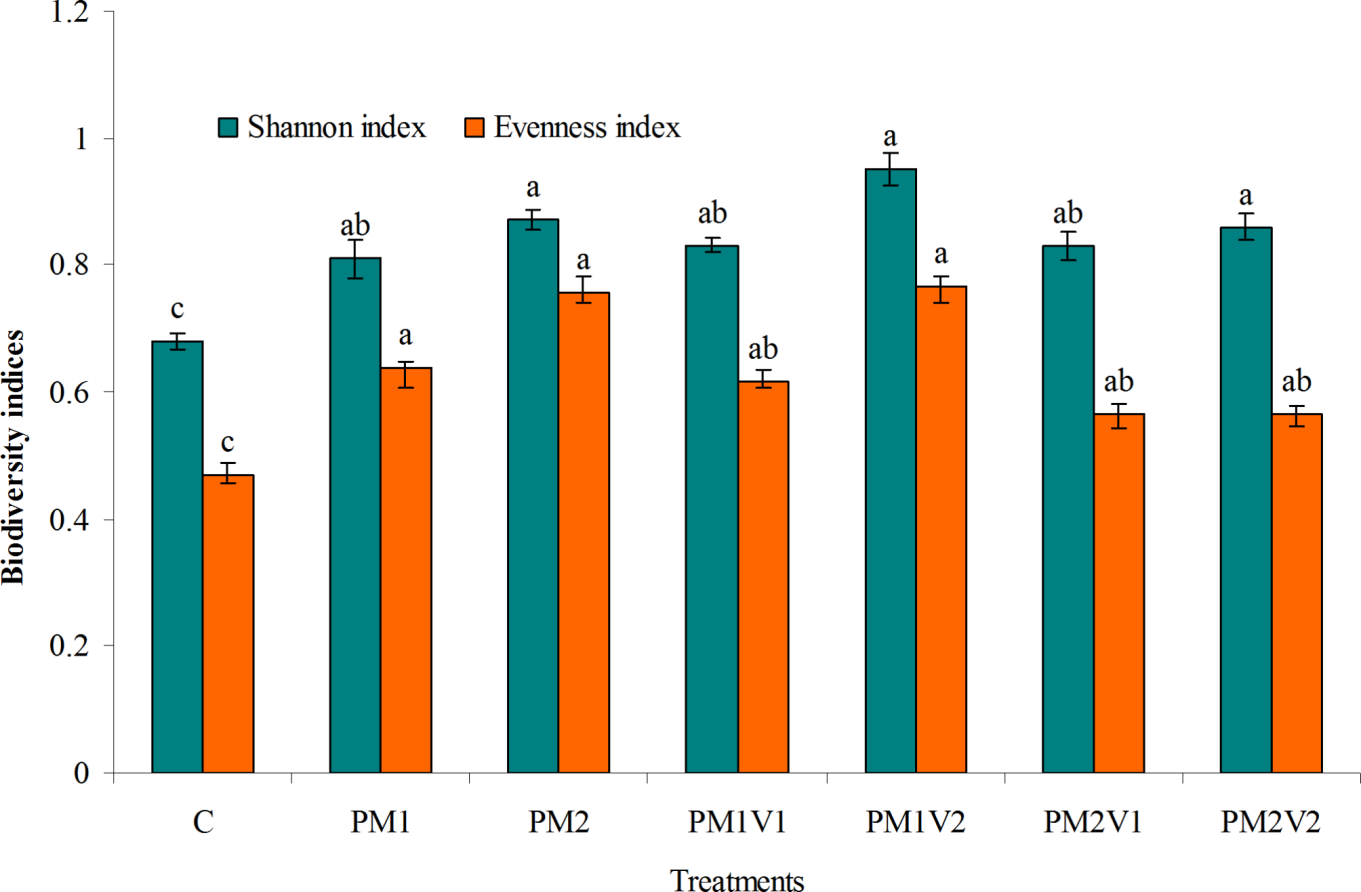
Effect of PM alone or co-applied with V on biodiversity indices. Similar letters indicate no significant variations among treatments

The Evenness index increased significantly with the addition of P alone or co-applied with V treatments versus the control in the mature stage (ripening stage) of barley growth, with the largest increase in evenness index recorded in the PM1V2 treatment and the lowest in the control (Fig. 2). The Evenness index increased from 0.486 to 0.637 in the PM1, and to 0.755 in the PM2. Evenness index increased by 26.95%, 63.67%, 16.26% and 17.08% in PM1V1, PM1V2, PM2V1 and PM2V2 relative to control, respectively.

**Fig. 3:**
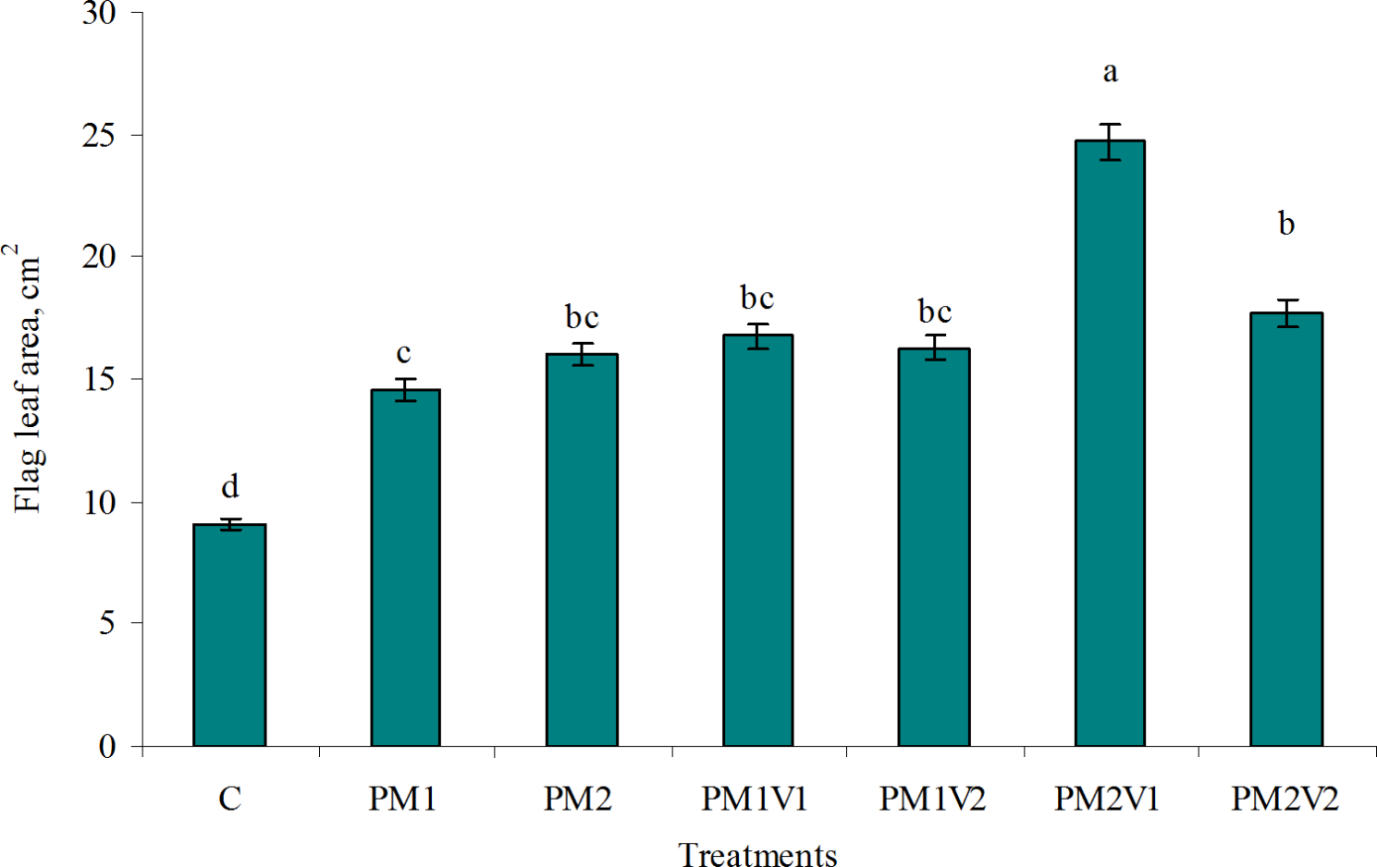
Effect of PM alone or co-applied with V on flag leaf area of barley plant. Similar letters indicate no significant variations among treatments

As shown in Fig. 3, in the pots treated with PM alone or mixed with V at different rates, Shannon’s index was significantly higher than that of the control treatment at the mature stage of barley growth. Shannon’s index increased by 19.11 and 27.94% by adding PM1 and PM2 compared with the control, respectively. Shannon’s index increased from 0.68 to 0.83 in PM1V1 and to 0.95 in PM1V2. The highest Shannon’s index was observed in PM2V1 pots, followed by treatments with PM2, PM2V2 and PM1V1 = PM2V1 and the lowest in the control.

### Effect of poultry manure and vinasse on barley productivity

The spike weight of barley was significantly increased in pots treated with PM alone or combined with V addition (Table 3). The spike weight increased by about 28.8 and 31.1% in the pots treated with PM1 and PM2 compared with the control, respectively. The spike weight increased from 14.02 to 19.81 g pot^− 1^ in the PM1V1, and to 18.52 g pot^− 1^ in the PM2V1. The application of PM1V1 gave the highest significant increase in the spike weight of barley plants compared to other treatments. A difference in spike weight of barley plants between the PM1 and PM2 as well as PM1V1 and PM2V2 is not statistically significant.

**Table 3:** Effects of PM alone or co-applied with V on growth of barley plants grown in calcareous soils. Results are expressed as mean ± standard deviation of five replicates

Treatments	Spike weight, g pot^− 1^	1000 grain weight g	grain weight g pot^− 1^	straw weight g pot^− 1^
C	14.02 ± 0.39 ^c^	35.60 ± 1.00 ^e^	5.76 ± 0.27 ^e^	89.23 ± 0.66 ^a^
PM1	18.07 ± 0.01 ^b^	42.40 ± 1.87 ^d^	7.08 ± 0.34 ^d^	73.04 ± 0.71 ^d^
PM2	18.38 ± 0.29 ^b^	48.43 ± 1.63 ^b^	9.90 ± 0.84 ^ab^	82.31 ± 0.71 ^b^
PM1V1	19.81 ± 0.23 ^a^	45.20 ± 1.35 ^c^	6.66 ± 0.07 ^d^	75.25 ± 0.92 ^c^
PM1V2	13.37 ± 0.33 ^d^	54.53 ± 0.72 ^a^	10.19 ± 0.26 ^a^	72.38 ± 1.13 ^de^
PM2V1	18.52 ± 0.69 ^b^	42.60 ± 0.44 ^d^	9.52 ± 0.17 ^b^	72.13 ± 0.26 ^de^
PM2V2	19.38 ± 0.26 ^a^	53.30 ± 0.72 ^a^	8.47 ± 0.14 ^c^	71.63 ± 0.26 ^e^

The straw weight of barley plants decreased significantly in the pots treated with PM manure alone or combined with V addition (Table 3). The straw weight decreased from 89.23 to 82.31 g pot^− 1^ in the PM2, and to 73.04 g pot^− 1^ in the PM1. Straw weight decreased as the rate of V increased. A difference in straw weight of barley plants between the PM1V2, PM2V1 and PM2V2 as well as PM1 is not statistically significant.

The application of PM alone or co-applied with V had a significant effect on the 1000-grain weight of barley (Table 3). In the soil treated with PM, 1000-grain weight of barley plants increased by 19.1 and 36.03% at 4.20 and 6.30 g kg^− 1^ soil, respectively. The 1000-grain weight increased from 35.6 to 45.20 g in the PM1V1, and to 54.53 g in the PM1V2. PM1V2 mixtures gave the best results in the 1000-grain weight of barley plants by 53.2% relative to the control.

The addition of PM alone or combined with V had a significant effect on the flag leaf area (FLA) (Fig. 2). In the pot amended with PM as compared to the control, the flag leaf area increased by 60.3 and 76.6% at 4.20 and 6.30 g kg^− 1^ soil, respectively. Flag leaf area of barley plants increased with the increase of PM addition. PM2V1 mixtures gave the best results in FLA by 172.6% relative to the control. The combined application of the PM and V amendments increased the area of the flag leaf compared to adding PM only. The difference in FLA of barley plants between the PM2, PM1V1, PM1V2 and PM2V2 treatments is not statistically significant.

The effect of adding PM alone or co-applied with V on the grain yield of the barley plant was significant (Table 3). The grain yield of the barley plant increased from 5.76 g pot^− 1^ in the control treatment to 9.52 and 10.19 g pot^− 1^ for the PM2V2 and PM2V1 treatments, respectively. The grain yield of the barley plant increased by about 22.9% and 71.8% in the pots treated with PM1 and PM2 compared with the control. PM2V1 mixtures gave the best results in grain yield by 76.90% compared to the control, although there were no significant differences between them and PM2. The difference in grain yield of barley plants between PM2 and PM2V1 is not statistically significant.

### Effect of organic amendments on soil fertility

The results of MBC and OM in soil increased significantly by adding PM only or mixed with V amendment compared to unamended soil (Table 4). The OM varied from 1.20% for control to 1.98% for PM2V2. When adding 6.30 g kg^− 1^ soil of PM to the soil, the MBC and OM increased by 70.8% and 22.5%, respectively, compared to the control. PM2V1 mixtures have the best results in OM with 65.0% compared to control, and V contributes more than 16% of them. The MBC and OM were higher in PM2V1 and PM2V2 than in the other treatment. Organic matter and MBC increased with increasing rates of PM and V addition. Additionally, differences in OM between PM1V2 and PM2V1 were not statistically significant. A difference in MBC between the PM1, PM2 and PM1V1 as well as PM1V2 and PM2V1 is not statistically significant.

**Table 4:** Effect of PM alone or co-applied with V on organic matter, soil microbial biomass (MBC) and availability of NPK. Results are expressed as mean ± standard deviation of five replicates

Treatments	O.M	MBC	K	P	N
	%	mg Kg^− 1^		mg Kg^− 1^	
C	1.20 ± 0.02^e^	2.16 ± 0.10^d^	248.19 ± 0.03^ g^	8.62 ± 0.03^ g^	26.46 ± 0.13^e^
PM1	1.47 ± 0.01^d^	3.15 ± 0.45^bc^	255.34 ± 0.02 ^f^	11.31 ± 0.05^f^	31.32 ± 0.06^d^
PM2	1.70 ± 0.02 ^c^	3.69 ± 0.13^b^	256.66 ± 0.31 ^e^	12.56 ± 0.02^d^	33.47 ± 0.22^c^
PM1V1	1.53 ± 0.04^d^	3.35 ± 0.08^bc^	268.60 ± 0.16^d^	12.28 ± 0.03^e^	36.90 ± 0.05^a^
PM1V2	1.77 ± 0.03^b^	5.37 ± 0.26^a^	280.45 ± 0.14^a^	13.85 ± 0.14^b^	35.34 ± 0.08^b^
PM2V1	1.77 ± 0.01^b^	5.44 ± 0.74^a^	270.60 ± 0.15^c^	13.41 ± 0.16^c^	36.78 ± 0.10^a^
PM2V2	1.98 0.02^a^	2.65 ± 0.57^ cd^	274.44 ± 0.02^b^	14.36 ± 0.00^a^	36.70 ± 0.17^a^

The application of PM alone or combined with V had a significant effect on the availability of nitrogen (N), phosphorus (P) and potassium (K) (Table 4). Availability of N, P and K increased with increasing rates of PM addition. High N and P availability were obtained in the PM2V2 and K in the PM1V2 compared to other treatments. The application PM1 and PM2 increased N and P availability by 18.37% and 26.49%, and 31.21% and 45.71%, respectively. The addition of PM increased the K availability from 248.19 ± 0.03 to 268.60 ± 0.6 mg kg^− 1^ in PM1V1 and to 280.45 ± 0.45 mg kg^− 1^ in PM1V2, respectively. A difference in availability PM between the PM1V1, PM2V1 and PM2V2 is not statistically significant.

## Discussion

**Fig. 4:**
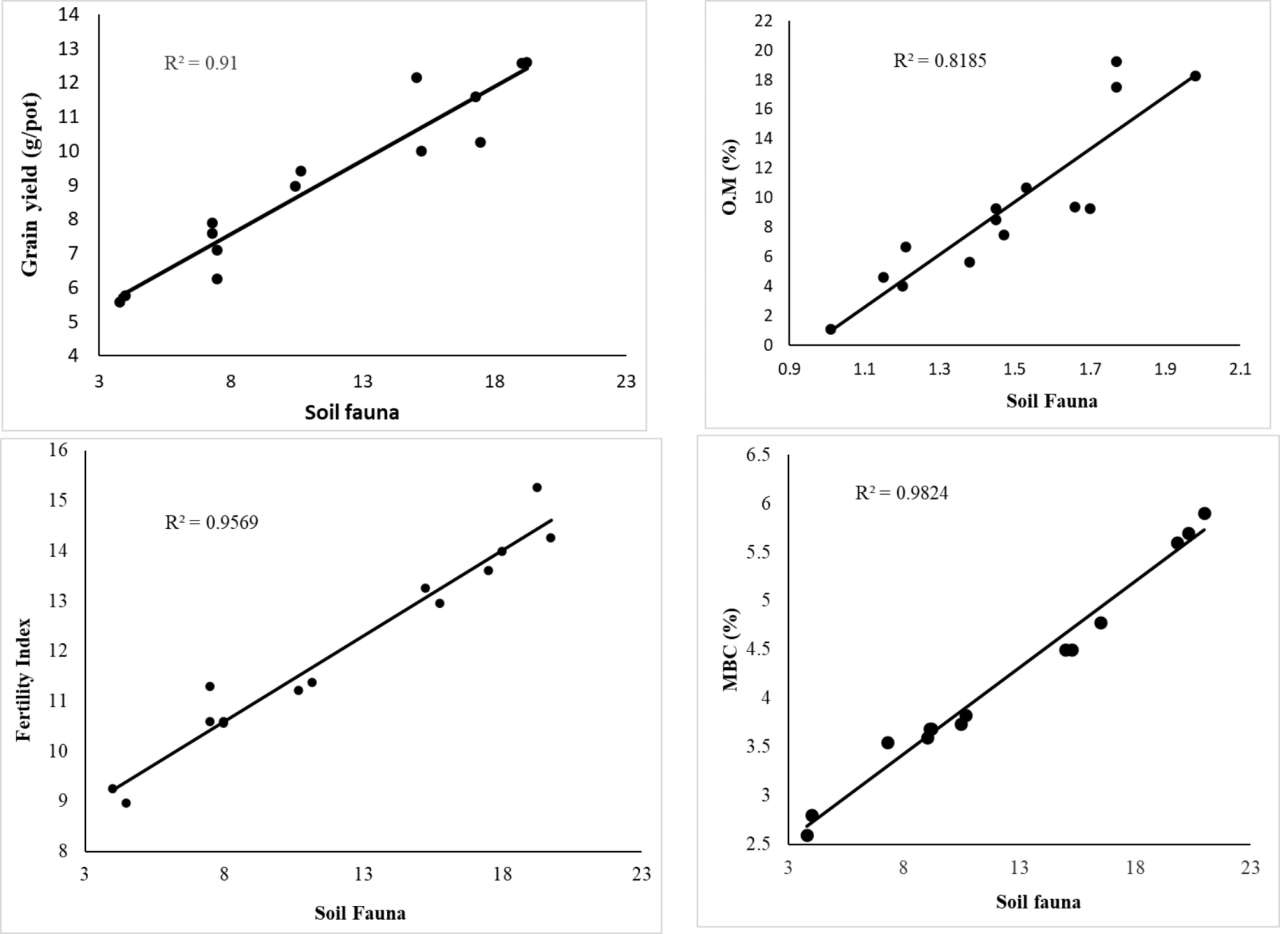
Correlation of soil fauna among the grain yield, organic matter, microbial biomass carbon, and soil fertility index after the PM addition alone or in combination with V

In this study, the total number of soil fauna increased significantly with the addition of organic amendments (PM alone or co-applied with V). The higher abundance of the total number of soil fauna in the soil treated with PM is due to the higher OM and other nutrient contents in the PM (Table 1). We found that soil treated with PM addition alone or in combination with V had a strong significant correlation between soil organic matter, soil fertility index, microbial biomass carbon and soil fauna (R^2^ = 0.82, P < 0.05), (R^2^ = 0.956, P < 0.05), and (R^2^ = 0.98, P < 0.05) (Fig. 4). Islam et al. [38] found that the strong positive correlation between the abundance of soil Collembola population with organic materials, phosphorus, potassium, nitrogen, as well as sulfur. Rosildaet al. [39] found that the populations of Acari and Collembola soils increased with increasing soil content of organic matter. The addition of organic matter to the soil has an effective effect in increasing the abundance and diversity of the total collembola and oribatid mites [40]. Wardle et al. [41] found that arthropod taxa increase with addition of organic matter as well as in organic farming systems. A significant effect of soil carbon on soil fauna has also been found in previous studies [42–44]. Furthermore, the carbon content of the roots greatly affected the species formation of Oribatida mites probably via rhizodeposition, as it feeds on dead roots or root-associated fungi [45]. Collembola were prevalent in this study suggesting that it benefited from organic matter coming from poultry manure and vinasse. The increase is due to the high rate of reproduction and the ability to adapt [46]. The high abundance of total number of soil fauna in the PM1V2 pots could be explained due to the high soil microbial biomass, organic matter and improved nutrient status in PM1V2 amended soil (Table 4). The application of manure compost has more influences on soil microbial community diversity and soil microenvironment [47]. The presence of ethanol and phenols in pots that received a high rate of V could be one of the reasons for the decrease in the total number of soil fauna. According to Wardle [48], vinasse and poultry manure form part of the plant’s natural defense system against infection and harmful microbial invasion. Low numbers of soil fauna in the control treatment, may be due to the absence of an external carbon source [49]. The abundance and diversity of fauna related to both soil fertility management and agro-ecological conditions in various fields [50].

Diversity indices for Shannon and Vennes were used to express biodiversity in soils treated with PM alone or co-applied with V, which showed a high value with their addition due to their high contents of nutrients and organic matter (Table 1). The increase in Shannon and Vennes indices with the addition of PM alone or combined with V was significant. Similar results were reported by Zhen et al. [51], who found that the highest value of the Shannon index in soil treated with organic manure and the lowest value in the N treatment in the maturity stage of maize.

Soil fertility is important for soil management because it reflects the production capacity of the soil to sustain growth of plants [52]. The increase in soil fertility with the addition of PM alone or mixed with V coincided with their higher content of OM, P, N, K and CEC (Table 1). Barley yield increased with the addition of V, which may be related to the higher content of vinasse of OM, K, Ca, N, and P (Table 1). You et al. [53] found that the application of V improves soil fertility and physical properties, and increases sugarcane yield without adverse effects on groundwater. Gemtos et al. [54] found that the yield of durum wheat increased by 32 or 46% with the addition of sugar beet V to the soil at a rate of 3500 or 7000 kg ha ^− 1^, respectively. Taha [55] attributed the increase in the growth and productivity of paspalum turf-grass after the application of V to the high percentage of potassium, nitrogen, zinc and molybdenum in it, and its improvement in the characteristics of the soil. These results are consistent with those obtained by Komdorfer and Anderson [56] on wheat, pigeon pea and maize [57], and on barley and spinach. Pedro et al. [58] found that the addition of PM increases wheat grain due to an increase in the number of tillers and grain. Winck et al. [59] note that the increase in collembolan species is working to higher C, P, S and N mineralization, which leads to an increase in the plant productively. Amujoyegbe et al. [60] reported an increase in leaf area, total chlorophyll content, and grain yield of maize and sorghum with the addition of PM. Jackson [61] reported that PM contains essential nutrients needed to enhance growth and productivity of crops. The addition of PM increases the soil’s organic matter content and improves its water holding capacity as well as its nutrient content, which enhances crop yield. Fagimi and Odebode [62] reported that PM applied at a rate of 10 t ha^− 1^ and 20 t ha^− 1^, increased plant height, number of leaves and fruit yield of peppers. It has been observed that the nutrients provided by poultry manure have positive effects on crop yield [63]. The data showed that the addition of PM in integration with V led to a significant increase in the growth and yield of barley. This increase may be due to the addition of organic fertilizers in the form of PM or V, which improves soil fertility by containing macro and micro nutrients, amino acids, organic acids, sugar and organic matter. Their addition also reduces soil pH, enhances the availability of nutrients and soil fauna, and ultimately improves yield components. PM and V improved the PM efficiency in ameliorating the soil fertility, which resulted in the best barley yield. We found that soil treated with PM addition alone or in combination with V had a strong significant correlation between soil fauna with MBC, grain yield of barley plants (R^2^ = 0.91 and R^2^ = 0.94, P < 0.05) (Fig. 4). Applying PM with V to soil can reduce fertilizer input, increase crop yield, plant nutrient uptake, and increase soil fauna.

## Conclusion

The results demonstrated that the addition of PM in integration with V led to a significant increase in the OM, MBC, availability of NPK, and yield of barley at different rates. Shannon’s index increased by 19.11% and 27.94% by adding PM1 and PM2, respectively compared to the control. In this study, the soil treated with PM addition alone or in combination with V had a strong significant correlation of soil fauna among the grain yield, organic matter, microbial biomass carbon, and soil fertility index. The interaction of PM and V at rates of 4.2 g kg^− 1^ and 6.3 g kg^− 1^ gave the best MBC and increased SOM content and soil fertility and thus increased plant growth as compared to adding PM alone. The results of this study propose the application of PM and V mixtures as a new sustainable strategy for growing barley in calcareous soils.

## Electronic supplementary material

Below is the link to the electronic supplementary material.

Supplementary Material 1

## Data Availability

The datasets used and analyzed during the current study are available from the corresponding author on reasonable request.
